# An introduction to the Marburg virus vaccine consortium, MARVAC

**DOI:** 10.1371/journal.ppat.1010805

**Published:** 2022-10-13

**Authors:** Robert W. Cross, Ira M. Longini, Stephan Becker, Karin Bok, David Boucher, Miles W. Carroll, Janet V. Díaz, William E. Dowling, Ruxandra Draghia-Akli, James T. Duworko, John M. Dye, Michael A. Egan, Patricia Fast, Amy Finan, Courtney Finch, Thomas R. Fleming, Joan Fusco, Thomas W. Geisbert, Anthony Griffiths, Stephan Günther, Lisa E. Hensley, Anna Honko, Ruth Hunegnaw, Jocelyn Jakubik, Julie Ledgerwood, Kerstin Luhn, Demetrius Matassov, Jeffrey Meshulam, Emily V. Nelson, Christopher L. Parks, Roxana Rustomjee, David Safronetz, Lauren M. Schwartz, Dean Smith, Paul Smock, Ydrissa Sow, Christina F. Spiropoulou, Nancy J. Sullivan, Kelly L. Warfield, Daniel Wolfe, Courtney Woolsey, Roland Zahn, Ana María Henao-Restrepo, César Muñoz-Fontela, Andrea Marzi

**Affiliations:** 1 Galveston National Laboratory, and Department of Microbiology and Immunology, University of Texas Medical Branch, Galveston, Texas, United States of America; 2 Department of Biostatistics, University of Florida, Gainesville, Florida, United States of America; 3 Institute for Virology, Philipps-Universität Marburg, Marburg, Germany; 4 Vaccine Research Center, National Institute of Allergy and Infectious Diseases, National Institutes of Health, Bethesda, Maryland, United States of America; 5 U.S. COVID-19 Response at U.S. Department of Health and Human Services, Washington, DC, United States of America; 6 Pandemic Sciences Institute, Nuffield Department of Medicine, Oxford University, United Kingdom; 7 World Health Organization, Geneva, Switzerland; 8 Coalition for Epidemic Preparedness Innovations (CEPI), Washington, Washington, DC, United States of America; 9 Johnson & Johnson—Global Public Health Research and Development, Spring House, Pennsylvania, United States of America; 10 Partnership for Research on Infectious Diseases in Liberia, Monrovia, Liberia; 11 Virology Division, United States Army Medical Research Institute of Infectious Diseases, Frederick, Maryland, United States of America; 12 Auro Vaccines, Pearl River, New York, United States of America; 13 IAVI, New York, New York, United States of America; 14 Sabin vaccine Institute, Washington, DC, United States of America; 15 University of Washington, Seattle, Washington, United States of America; 16 Public Health Vaccines, Cambridge, Massachusetts, United States of America; 17 National Emerging Infectious Diseases Laboratories, Boston University School of Medicine, Boston, Maryland, United States of America; 18 Bernhard Nocht Institute for Tropical Medicine, Hamburg, Germany; 19 Integrated Research Facility, Division of Clinical Research, National Institute of Allergy and Infectious Diseases, National Institutes of Health, Frederick, Maryland, United States of America; 20 Immune Biology of Retroviral Infection Section, Vaccine Branch, National Cancer Institute, National Institutes of Health, Bethesda, Maryland, United States of America; 21 Janssen Vaccines & Prevention, Leiden, the Netherlands; 22 Zoonotic Diseases and Special Pathogens Division, National Microbiology Laboratory, Public Health Agency of Canada, Winnipeg, Manitoba, Canada; 23 Bacterial and Combination Vaccines, Public Health Agency of Canada, Ottawa, Ontario, Canada; 24 Collaborative Clinical Research Branch, Division of Clinical Research, National Institute of Allergy and Infectious Diseases, National Institutes of Health, Rockville, Maryland, United States of America; 25 Viral Special Pathogens Branch, Division of High-Consequence Pathogens and Pathology, National Center for Emerging and Zoonotic Infectious Diseases, Centers for Disease Control and Prevention, Atlanta, Georgia, United States of America; 26 Emergent BioSolutions, Gaithersburg, Maryland, United States of America; 27 Laboratory of Virology, Division of Intramural Research, National Institute of Allergy and Infectious Diseases, National Institutes of Health, Hamilton, Montana, United States of America; University of Iowa, UNITED STATES

## Abstract

The emergence of Marburg virus (MARV) in Guinea and Ghana triggered the assembly of the MARV vaccine “MARVAC” consortium representing leaders in the field of vaccine research and development aiming to facilitate a rapid response to this infectious disease threat. Here, we discuss current progress, challenges, and future directions for MARV vaccines.

## Introduction

On August 3, 2021, the World Health Organization (WHO) was notified of a confirmed case of Marburg virus (MARV) disease (MVD) in the Guéckédou prefecture in Guinea, with 173 contacts, including 14 high-risk contacts based on exposure [1]. This was the first time MARV was detected in the country and raised alarms about a possible outbreak expansion that may lead to an epidemic such as the Ebola virus (EBOV) disease (EVD) epidemic in West Africa in 2013 to 2016. Fortunately, no additional MVD cases were detected in Guinea and the end of the outbreak was declared on September 16 [2].

On July 7, 2022, WHO reported 2 suspected cases of MVD in the southern Ashanti region of Ghana and received diagnostic confirmation for MARV on July 15, 2022. On July 25, 2022, 2 additional cases were reported of which only one was confirmed to be MVD bringing the current case count to 3. Approximately 180 contacts are currently being followed in the Ashanti, Western, and Savannah regions of Ghana [3]. This continued reemergence of MARV highlights the need for vaccines to prevent future MVD outbreaks.

To foster the rapid development of MARV vaccines, the WHO R&D Blueprint has convened a group of experts with the aim to promote preclinical and clinical development of MARV vaccine candidates. This WHO-coordinated consortium for the development of MARV vaccines (MARVAC; Box 1) is based on the same sharing principles that governed the scientific interactions of the WHO COVID-19 working groups. In this article, we summarize the current state of MARV medical countermeasures and provide an outlook of the role of MARVAC on accelerating vaccine evaluation and approval.

**Box 1. The MARVAC**

MARVAC is a WHO-coordinated consortium to promote international collaboration for the development of MVD vaccines. The consortium builds on the previous success of WHO working groups on COVID-19 preclinical models and assays that accelerated the development of COVID-19 vaccines by rapidly sharing scientific findings and protocols. This consortium includes shareholders from industry, nonprofit organizations, government, and academia in order to maximize its effectiveness. This joint venture is supported by the following principles: (1) sharing of assays and reagents; (2) promoting access to laboratory networks in MVD-endemic countries; and (3) promoting structural support for preclinical development of upcoming MVD vaccine and therapeutic candidates.

### Marburg virus disease

MARV was identified in 1967 as the causative agent of a hemorrhagic disease outbreak in Marburg and Frankfurt, Germany, with the outbreak originating from nonhuman primates (NHPs) imported from Africa [4]. Since then, outbreaks of MVD have sporadically occurred throughout Africa with the largest outbreak recorded in 2004 to 2005 in Angola with 252 cases and an 88% case fatality rate [5]. The recent identification of the MVD cases in Guinea and Ghana are the consequence of reinforced laboratory networks and diagnostics capacity that were implemented after the EVD epidemic. Most of the identified MVD patients in Guinea and Ghana died of the disease, with MARV confirmed as the causative agent retrospectively [3,6,7].

NHP studies have been performed with several different MARV isolates with most using either the MARV-Musoke (Kenya, 1980; [8]), MARV-Angola (Angola, 2005; [9]), or Ravn virus (RAVV; Kenya, 1987; [10]) isolates. RAVV is genetically distinct form MARV and regarded as its own entity within the *Marburg marburgvirus* species. Infections with 1,000 plaque-forming units by the intramuscular route of MARV-Musoke, MARV-Angola, and RAVV are uniformly lethal in cynomolgus macaques; however, disease progresses fastest with MARV-Angola resulting in death within 9 days [11].

To date, there are no regulatory agency-approved MARV vaccines or therapeutics. While filovirus diseases are of great public health consequence, limited financial support from the public sector to fund the development of medical countermeasures existed prior to the 2013 to 2016 EBOV epidemic. However, after 2016, basic and translational filovirus research has been increased through biodefense and research grants, which has helped to advance licensing of EBOV vaccines and treatments for outbreak control [12–14]. For MARV, this funding allowed for the expansion of animal models and countermeasure strategies for preclinical evaluation. Several approaches have shown promise in NHP studies, with vaccines as well as antivirals advancing into clinical development, including Phase I clinical trials.

### Animal models

Countermeasure development requires efficacy testing in animal models that recapitulate hallmark features of human clinical disease, which can serve as a predictive measure for the potential benefit of a drug or vaccine. Rodent models require virus adaptation, but are the preferred screening models due to availability, cost, and space limitations as all infectious work has to be performed in maximum containment laboratories. The “gold standard” animal model for MARV are NHPs, particularly cynomolgus and rhesus macaques, due to their close recapitulation of clinical disease in humans and uniform lethality [15]. Most vaccine efficacy studies have been performed in cynomolgus macaques, whereas rhesus macaques are more commonly used for treatment studies [8,16].

### Vaccines

MARV vaccine development started soon after the discovery of the virus with limited success [17,18]. Numerous different vaccine platforms have been evaluated for MARV in rodent models [18]; however, only a portion of these candidate vaccines demonstrated protective efficacy in NHPs and only these are shown in Table 1. The MARV glycoprotein (GP) is the main antigen used in all successful candidate vaccines and confers protection against multiple strains of MARV and RAVV (Table 1). Vaccine approaches for MARV include multidose, single-dose, fast-acting, live-attenuated nonreplicating, and replicating viral vector vaccine regimens. While fast-acting, single-dose vaccines would be deployed in reactive vaccination campaigns during outbreaks, routine vaccination of at-risk populations could be achieved with several candidate vaccines. Regardless, the need for boosters will be determined by the durability of the acquired immunity induced by the respective vaccine.

**Table 1:** MARV vaccines with protective efficacy in the preclinical NHP model.

Vaccine	Challenge Virus	Vaccine doses	Time between doses [d]	Time to Challenge [d]*	Survival [%]	Developer	Ref.	
**Whole Virus**								
**inact. MARV**	MARV Popp	2	14	21	50		[18]	
**Subunit**								
**VLPs + adjuvant**	MARV Musoke	3	42	28	100		[18]	
**VLPs + adjuvant**	MARV Musoke, Angola	3	42	28	100	USAMRIID	[18]	
**MARV GP + adjuvant**	MARV Angola	3	21	28	100		[39]	
**MARV GP, EBOV GP + adjuvant**	MARV Angola	3	21	28	100		[39]	
**DNA**								
**MARV GP**	MARV Musoke	3	28	28	67	USAMRIID	[18]	
**MARV GP**	MARV Angola	4	3× 28, then 105	21	100	NIAID	[18]	
**MARV GP, RAVV GP, EBOV GP, SUDV GP**	MARV Musoke	3	28	56	100	USAMRIID	[18]	
**DNA + rec. Adenovirus**								
**3x DNA-MARV GP, 1x rAd5-MARV GP**	MARV Angola	4	28 DNA, then 84 rAd5	42	100	NIAID	[18]	
**Replicon**								
**VEEV-MARV GP, VEEV-MARV NP, both**	MARV Musoke	3	28	35	67–100	USAMRIID	[18]	
**rec. Adenovirus**								
**CAdVax-panFilo**	MARV Musoke, Ci67	2	63	42 or 112	100		[18]	
**rAd5-MARV GP**	MARV Angola	1	n/a	28	100		[18]	
**rAd26-MARV GP + rAd35-MARVGP**	MARV Angola	2	28	28	100	Janssen	[19]	
ChAd3-MARV	MARV Angola	1	n/a	7, 14, 21, 28, 35, or 185	100	Sabin	[21]
ChAd3-MARV	MARV Angola	1	n/a	365	75	Sabin	[21]
**rec. Vesicular stomatitis virus**							
**VSV-MARV**	MARV Musoke, Popp	1	n/a	28 or 113	100	PHAC, IAVI	[18]
**VSV-MARV**	MARV Musoke, Angola, RAVV	1	n/a	28	100	PHAC, IAVI	[18]
**VSV-MARV**	MARV Angola	1	n/a	28	100	PHAC, IAVI	[18]
**VSV-EBOV, VSV-SUDV, VSV-MARV**	MARV Musoke	1	n/a	28 or 59	100	PHAC, IAVI	[18]
**VSV-MARV**	MARV Musoke	1	n/a	407	100	PHAC, IAVI	[18]
**VSV-MARV**	MARV Angola	1	n/a	35	100	PHAC, PHV	[27]
**VSV-MARV**	MARV Angola	1	n/a	14 or 7	100	PHAC, PHV	[28]
**VSV-MARV**	MARV Angola	1	n/a	3	75	PHAC, PHV	[28]
**VSV-N4CT1-MARV**	MARV Angola	1	n/a	7	100	Auro Vaccines	[25]
**VSV-N4CT1-MARV**	MARV Angola	1	n/a	5	80	Auro Vaccines	[25]
**VSV-N4CT1-MARV**	MARV Angola	1	n/a	3	20	Auro Vaccines	[25]
**Trivalent VesiculoVax**	MARV Angola	1	n/a	28	100	Auro Vaccines	[24]
**Quadrivalent VesiculoVax**	MARV Angola	2	56	28	100	Auro Vaccines	[23]

*Time until challenge after vaccination was completed.

n/a, not applicable.

One adenovirus (Ad)-based vaccine is being developed by Janssen and is based on an Ad26 vector encoding the MARV Angola GP [19]. This vaccination strategy follows the European Medicines Agency (EMA)-approved EBOV prime/boost vaccine Zabdeno/Mvabea (Ad26-ZEBOV/MVA-BN-filo) developed by this company. In preclinical studies for MVD, prime-boost vaccination with Ad26-MARV/Ad35-MARV showed protection against MARV-Angola, and durable antibody responses [19]. However, only the Ad26-MARV vaccine will be moved forward. Clinical trials could evaluate the immunogenicity of a single-dose vaccine and explore if a boost vaccination with the EMA-approved vector Mvabea (MVA-BN-Filo) [20] or another Ad-vectored filovirus vaccine is beneficial. Currently, there are 4,500 clinical grade doses of the Ad26-MARV vaccine available for emergency use.

Another Ad-based vaccine is a single-dose vaccine being developed by the Sabin Vaccine Institute that uses a chimpanzee Ad (ChAd) vector to circumvent antivector immunity. In NHPs, this ChAd3-MARV vaccine provides both rapid (within 1 week) and longer-term (6 to 12 months) protection against lethal MARV challenge (Table 1) [21]. A Phase I clinical trial with 40 participants was conducted comparing 2 doses of the vaccine, 10^10^ versus 10^11^ particle units [22]. The vaccine showed a favorable safety profile with no reported severe adverse events. MARV GP-specific immunoglobulin G (IgG) was detected in 80% to 90% of the participants after 28 days with the higher dose vaccination resulting in a higher IgG titer (660.7 versus 392.7 units, respectively). Overall, the induced immune responses are in the range found to correlate with protection in NHP studies [21]. Phase II clinical trials in Africa are planned for 2023. Clinical grade ChAd3-MARV has been vialed and tested. Approximately 450 of these doses are immediately available for outbreak response, and additional drug substance (approximately 18,000 doses) is available for vialing.

The first of 3 vesicular stomatitis virus (VSV)-based vaccines described here is the attenuated VSV-N4CT1-MARV vaccine developed by Auro Vaccines. It is a VSV full-length vector encoding a VSV N gene translocation together with a truncation of the VSV G protein cytoplasmic tail and the MARV-Angola GP as viral antigen. Two doses have been shown to uniformly protect NHPs in preclinical studies in a trivalent (EBOV GP, Sudan virus (SUDV) GP, MARV GP) or quadrivalent (trivalent + Lassa virus GP) vaccine formulation, with the trivalent version also showing full protection as a single dose (Table 1) [23,24]. In addition, a single dose of the monovalent vector is protective within 7 days (Table 1) [25]. Clinical data for the single-dose MARV vaccine candidate are not yet available; however, manufacturing of clinical grade material is planned for late 2022.

The remaining 2 VSV-based vaccines both originate from the attenuated VSVΔG vector in which the VSV-G is replaced by the MARV GP. This strategy follows the example of the US FDA- and EMA-approved EBOV vaccine Ervebo by Merck. The International AIDS Vaccine Initiative (IAVI) is developing the VSV-MARV Musoke vector, and Public Health Vaccines (PHV) the VSV-MARV Angola vector. Both vectors have shown uniform protection after a single-dose vaccination in the NHP model of infection against both MARV and RAVV (Table 1) [26–28]. Based on clinical data for Ervebo [29,30], vaccination with either of the VSV-MARV vectors is expected to provide protection with limited adverse effects. IAVI will manufacture clinical grade material in late 2022 (approximately 2,000 vials); clinical grade material of VSV-MARV by PHV has been manufactured as is available for clinical testing.

### Vaccines clinical trial design

Supported by the success of a ring vaccination trial for EBOV carried out in Guinea [29], efficient and reliable randomized Phase III evaluations of efficacy trials for MARV candidate vaccines listed in Table 1 should be conducted. The MARVAC is developing clinical trial designs that will accommodate the complex epidemiology of MARV transmission by assessing evidence accumulated over multiple outbreaks in time and location [31]. Likely, a number of feasible trial designs will be developed according to the format specified by the WHO Blueprint for emerging infectious disease threats [32]. The trials will be double blinded whenever possible. The best comparators will be either vaccine placebos or active comparator vaccines, not related to the MARV protection, with benefit to the population, e.g., hepatitis A vaccine. If this is not possible, then delayed vaccination will be performed. There will be 3 categories of possible trial designs [33,34]. The overall platform trial will allow use of any of these categories, as well as the aggregation of evidence when more than one has been used [35]. In the absence of an outbreak, disease endpoints are not possible and trials designs will be adapted to fit regulatory requirements.

### Therapeutics

Compared to preclinical and clinical evaluation of EBOV therapeutics, the development of MARV therapeutics has evolved at a much slower pace. As with EBOV, antiviral efficacy against MARV infection in NHPs has historically served as the benchmark to rate predictive efficacy in humans and justify subsequent clinical trial efforts. Several promising approaches ranging from pan-filoviral small molecule antivirals to MARV-specific monoclonal antibody approaches or even combinations of the two have shown impressive postexposure efficacy in NHPs at late-stage disease (Table 2). These therapeutic approaches may be ideal for further development for use in humans. Furthermore, postexposure vaccine approaches have shown promise against MVD. A recent adaptive clinical trial in the Democratic Republic of Congo [36] has fortified evaluation criteria and allowed for recent approval of immunotherapeutics against EBOV. This approach [37,38] may serve as an ideal framework to navigate initiation of human trials for MARV exposures in concert with guidance from the MARVAC.

**Table 2:** MARV therapeutics with protective efficacy in the NHP model.

Treatment	Challenge virus	Treatment postchallenge	Treatment dose	Number of doses	Survival [%]	Ref.
**VSV-MARV**	MARV Musoke	20–30 minutes	1 × 10^7^ PFU	1	100	[40]
	MARV Musoke	1 day	2 × 10^7^ PFU	1	83	[40]
	MARV Musoke	2 days	2 × 10^7^ PFU	1	33	[40]
	MARV Angola	20–30 minutes	1 × 10^3^ PFU	1	25	[41]
	MARV Angola	20–30 minutes	50 PFU	1	89	[42]
**VSVN2CT1-MARV**	MARV Angola	20–30 minutes	50 PFU	1	80	[42]
**VSVN4CT1-MARV**	MARV Angola	20–30 minutes	50 PFU	1	60	[41]
**MR191-N**	MARV Angola	4 days	50 mg/kg	2	100	[40]
	MARV Angola	5 days	50 mg/kg	2	80	[40]
	RAVV	5 days	50 mg/kg	2	100	[40]
**MR186-YTE**	MARV Angola	5 days	100 mg/kg	1	100	[43]
		6 days	100 mg/kg	1	0	[43]
**MR186-YTE + Remdesivir**	MARV Angola	6 days	100 mg/kg	1	80	[43]
	MARV Angola	6 days	10 mg/kg load. 5 mg/kg maint.	12	80	[43]
**Remdesivir**	MARV Angola	5 days	10 mg/kg load. 5mg/kg maint.	12	80	[43]
	MARV Angola	6 days	10 mg/kg load. 5 mg/kg maint.	12	0	[43]
	MARV Angola	4 or 5 days	10 mg/kg load. 5 mg/kg maint.	12	85	[44]
	MARV Angola	5 days	5 mg/kg load. 5 mg/kg maint.	12	50	[44]
**BCX4430**	MARV Musoke	1 hour	15 mg/kg	30	83	[40]
	MARV Musoke	1 day	15 mg/kg	28	100	[40]
	MARV Musoke	2 days	15 mg/kg	26	100	[40]
**siRNA (NP)**	MARV Angola	1, 2, 3, or 4 days	0.5 mg/kg	7	100	[40]
	MARV Angola	5 days	0.5 mg/kg	7	50	[40]
	RAVV	3 or 6 days	0.5 mg/kg	7	100	[40]
**PMO*plus* (pool)**	MARV Musoke	30–60 minutes	40 mg/kg	14	100	[40]
**PMO*plus* (NP)**	MARV Musoke	1 hour	15 mg/kg	14	83	[40]
	MARV Musoke	1 or 4 days	15 mg/kg	14	83	[40]
	MARV Musoke	2 days	15 mg/kg	14	100	[40]
**rNAPc2**	MARV Angola	10 minutes	30 μg/kg	15	17	[40]
**IFNβ**	MARV Musoke	1 hour	35 μg/kg	15	33	[40]
**Favipiravir**	MARV Angola	at challenge	250 mg/kg load. 150 mg/kg maint.	3	85	[45]

load., loading dose; maint., maintenance dose.

## Conclusions

With the MDV cases in Guinea and Ghana, outbreaks of this filovirus disease have now been reported across the entire sub-Saharan Africa region. With case fatality rates of up to 88% and the lack of licensed medical countermeasures, there is an urgent need for action to accelerate the evaluation and approval of MVD vaccines and therapeutics. Following the inception of the WHO-coordinated MARVAC, we have presented here the current status of the most advanced MVD medical countermeasures. We highlight the need for global research coordination, including sharing of assays and continuous efforts on capacity building among private and public sectors. Increased surveillance and diagnostics are also required for early detection of future outbreaks as well as rapid deployment of vaccines and therapeutics.
